# Anti-malarial resistance in Mozambique: Absence of *Plasmodium falciparum* Kelch 13 (K13) propeller domain polymorphisms associated with resistance to artemisinins

**DOI:** 10.1186/s12936-023-04589-0

**Published:** 2023-05-19

**Authors:** Clemente da Silva, Daniela Matias, Brigite Dias, Beatriz Cancio, Miguel Silva, Ruben Viegas, Nordino Chivale, Sonia Luis, Crizolgo Salvador, Denise Duarte, Paulo Arnaldo, Sonia Enosse, Fatima Nogueira

**Affiliations:** 1 Global Health and Tropical Medicine (GHTM), Department of Medical Parasitology, Instituto de Higiene e Medicina Tropical, Universidade Nova de Lisboa, Rua da Junqueira Nº100, 1349-008 Lisbon, Portugal; 2 Instituto Nacional de Saúde (INS), Av. Eduardo Mondlane Nº 1008, Caixa Postal 264, Maputo, Mozambique; 3 Hospital Provincial de Matola, 2CPV+55, Matola, Mozambique; 4 Malaria Consortium, Av. Lucas Elias Kumato, Nº 118. Bairro da Sommershield, Maputo, Mozambique

## Abstract

**Background:**

Malaria remains one of the most serious public health problems in sub-Saharan Africa and Mozambique is the world's fourth largest contributor, with 4.7% of disease cases and 3.6% of total deaths due to malaria. Its control relies on the fight against the vector and treatment of confirmed cases with anti-malarial drugs. Molecular surveillance is an important tool for monitoring the spread of anti-malarial drug resistance.

**Methods:**

A cross-sectional study recruited 450 participants with malaria infection detected by Rapid Diagnostic Tests, from three different study sites (Niassa, Manica and Maputo) between April and August 2021. Correspondent blood samples were collected on filter paper (Whatman® FTA® cards), parasite DNA extracted and *pfk13* gene sequenced using Sanger method. SIFT software (Sorting Intolerant From Tolerant) was used, predict whether an amino acid substitution affects protein function.

**Results:**

No *pfkelch13*-mediated artemisinin resistance gene mutation was detected in this study settings. However, non-synonymous mutations were detected at prevalence of 10.2%, 6% and 5% in Niassa, Manica and Maputo, respectively. Most (56.3%) of the reported non-synonymous mutations were due to substitution at the first base of the codon, 25% at the second base and 18.8% at the third base. Additionally, 50% of non-synonymous mutations showed a SIFTscore bellow cut off value of 0.05, therefore, they were predicted to be deleterious.

**Conclusion:**

These results do not show an emergence of artemisinin resistance cases in Mozambique. However, the increased number of novel non-synonymous mutations highlights the relevance of increasing the number of studies focused on the molecular surveillance of artemisinin resistance markers, for its early detection.

## Background

Malaria remains one of the most serious public health problem in sub-Saharan Africa [1] and Mozambique is the world's fourth largest contributor, with 4.7% of disease cases and 3.6% of total deaths due to malaria [2]. In 2021, the World Health Organization (WHO) recommended the use of a malaria vaccine to protect children, however, the vaccine has low efficacy and its distribution is not yet widespread in Mozambique [3]. Therefore, malaria control relies on the fight against the vector and the administration of anti-malarial drugs [4].

*Plasmodium falciparum*, the most virulent of the five species that infect humans, has developed resistance to the successively introduced anti-malarial drugs, including to the currently recommended artemisinin-based combination therapy (ACT) introduced in Mozambique in 2009 [5, 6]. Artemisinin and its derivatives play an important role in killing *P. falciparum* by inhibiting the activity of phosphatidylinositol-3-kinase (PfPI3K) [7, 8] and like any other drug, it may have side effects, but they are poorly expressed [7, 9, 10]. Historically, cases of anti-malarial resistance have their starting point in Southeast Asia [6, 11], move through east Africa’s coast and spread to the rest of the continent [12–16]. In Mozambique, ACT using artemether–lumefantrine (AL) or artesunate–amodiaquine (AS–AQ) is currently the first-line treatment for uncomplicated malaria [1, 17–21].

Mutations in the *pfk13* gene have been correlated with delayed parasite clearance (tolerance) after administration of ACT [7, 21]. Some of the mutations are validated single nucleotide polymorphisms (SNPs), used as molecular markers for the surveillance of artemisinin-resistant malaria parasites (F446L, N458Y, M476I, Y493H, R539T, I543T, P553L, R561H, P574L, C580Y) (WHO, 2019). Recently, *P. falciparum* parasites carrying the validated SNPs F446I, M476I, P553L, R561H, P574L, C580Y and A675V have emerged and expanded or been identified in Africa [22], in Angola, Ghana [23], Mali, Rwanda [24–27]. In Mozambique, available studies on the prevalence of *pfK13* SNPs, refer to parasite samples collected prior to 2018 [28–31]. Thus, the present study was designed to update the current profile of SNPs in *P. falciparum pfk13* associated with artemisinin resistance in 3 provinces (Niassa, Manica and Maputo) of Mozambique.

## Methods

### Ethical considerations

The study protocol obtained ethical clearance by the National Bioethics Committee for Health of Mozambique (CNBS—IRB00002657) (Ref: 131/CNBS/2021) dated: March 2021.

### Study settings and sample collection

**Fig. 1:**
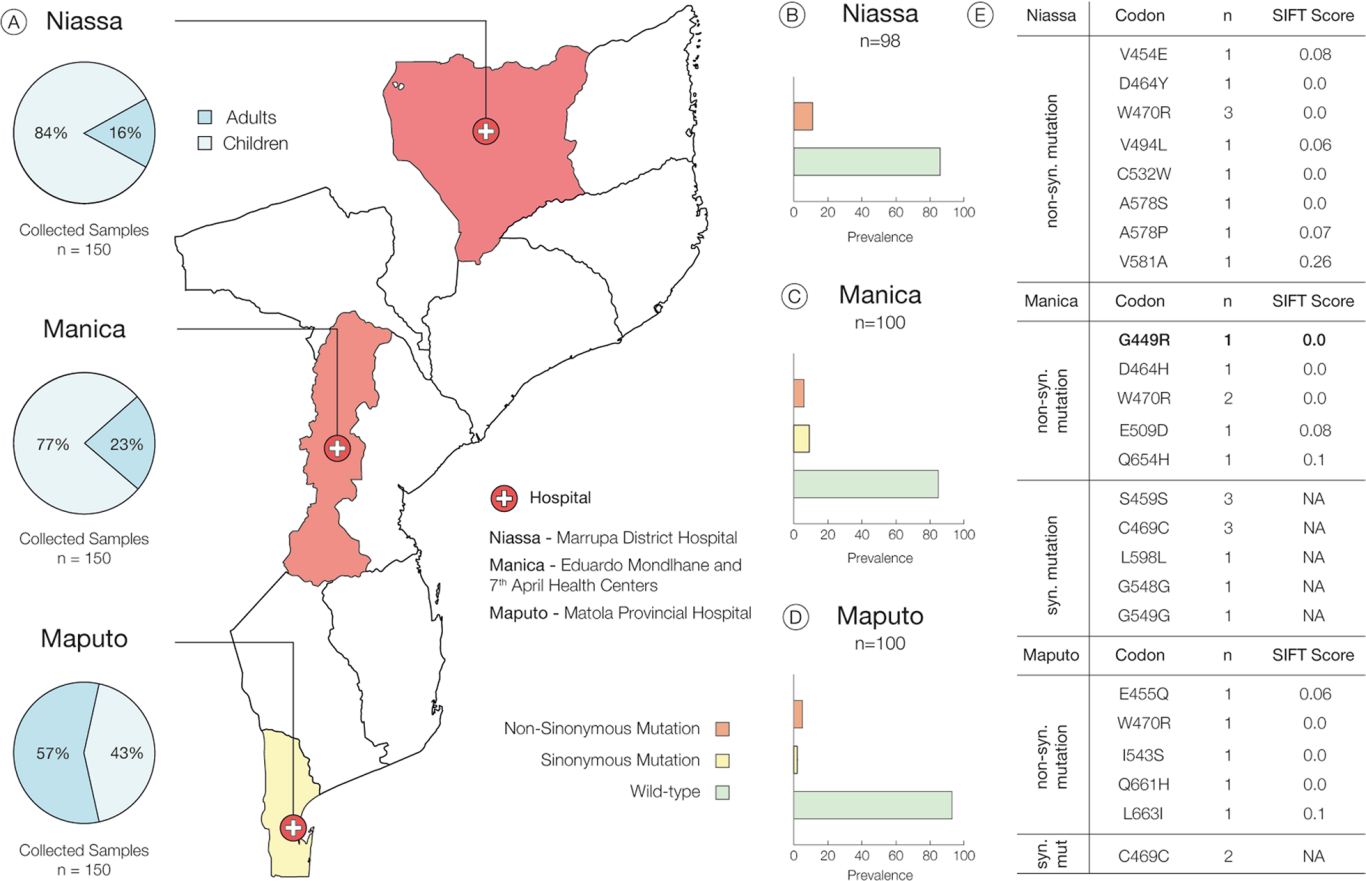
*Plasmodium falciparum* k13 polymorphisms profile in Mozambique. **A** Study sites and sample size. n-sample size in each province. Pie chart depict the percentage of children (Beau blue) and adults (Steel blue) enrolled in the study. **B**–**D** Prevalence of *pfk13* mutations in Niassa, Manica, and Maputo, respectively. Non-synonymous mutation (orange), synonymous mutation (yellow) and Wild type (green). **E** Mutations in 3 provinces and the respective codons and SIFT scores. Syn- Synonymous; Non.Syn- Non-synonymous. The candidate marker codon reported as G449A instead of G449R observed in our analyses (in bold). Figure was created using Illustrator version 26.3

Malaria is endemic throughout Mozambique, ranging from hyper-endemic areas along the coastline, meso-endemic areas in the interior lowlands and some hypo-endemic areas in the interior highlands. Several factors contribute to this endemicity, ranging from climatic and environmental conditions, such as favourable temperatures and rainfall, as well as favourable breeding sites for the vector. Most of the country has year-round throughout the year, with peaks during the rainy season from December to April [4]. Patient recruitment took place in 3 different epidemiological settings, in northern (Hospital distrital de Marrupa in Niassa), central (*Centro de Saúde Eduardo Mondlhane* and *Centro de Saúde 7 de abril* in Manica) and southern (*Hospital provincial da Matola* in Maputo) area of Mozambique, between April and August of 2021. One hundred and fifty samples were collected in each study site (Fig. 1A). The choice of Niassa, Manica and Maputo provinces not only is aligned with the study scientific goals but also with the objectives of *Instituto Nacional de Saúde*, and National Malaria Control Programme Mozambique, contributing to the systematic mapping of malaria cases at the national level.

A total of 450 participants of all ages with malaria positive Rapid Diagnostic Test (RDT) were recruited and provided 100 μL of blood samples on filter papers (Whatman® FTA® cards), after written informed consent. All dried blood spot samples were then stored under − 20 °C until they were used for genotyping.

### Characterization of* Pfk13* gene polymorphisms

Parasite genomic DNA from dried blood spots was extracted using the Chelex method [32], and DNA was stored at − 20 °C. Real-time PCR was used for *P. falciparum* confirmation. PCR reactions targeting the 18S rRNA gene were conducted as described in Rosanas-Urgell et al. 2010, with modifications. Briefly, forward primer 5′-TATTGCTTTTGAGAGGTTTTGTTACTTTG-3′ and reverse primer ACCTCTGACATCTGAATACGAATGC and the probe FAM-ACGGGTAGTCATGATTGAGTT-MGB-BHQ were used. PCR reaction mixture consisted of 7.5 μL of 2X (NZYTECH, Portugal), 600 nM of each primer and 200 nM of FAM™-labeled probe (IDT Integrated DNA Technologies, USA), 1 μL of genomic DNA and water up to 15 μL. PCR conditions: 50 °C for 2 min and 95 °C for 10 min; these were followed by 40 cycles at 94 °C for 30 s and a final cycle at 60 °C for 1 min. Triplicate samples were assayed in the Bio-Rad 500 Real Time PCR System™ (Applied Biosystems, USA). All reactions were performed with positive controls (DNA from 3D7 strain of *P. falciparum* culture).

The *Pfk13* fragment, containing the main polymorphisms associated with resistance to artemisinin, was amplified by nested PCR as described by Escobar and co-workers [29], with slight adjustements. Briefly, specific primers were developed for this purpose (forward—5′-CTATACCCATACCAAAAGATTTAAGTG-3′, reverse—5′-GCTTGGCCCATCTTTATTAGTTCCC-3′), obtaining a fragment of 902 bp (from codon 412 to codon 723). PCR conditions [33]: 94 °C 3 min; [94 °C 30 s, 57 °C 30 s, 72 °C 30 s] 10×; [94 °C 30 s, 55 °C 30 s, 72 °C 30 s] 30×; 72 °C 3 min. PCR products were analysed by electrophoresis on a 2% agarose gel stained with GreenSafe Premium (Nzytech, Portugal) to confirm amplification of targeted fragments. All positive PCR products were then purified using SureClean Plus (Bioline) and shipped to Eurofins Genomics (GATC services, Germany), to proceed with Sanger sequencing. Successfully sequenced samples were aligned to the PF3D7_1343700 gene using Multalign software (http://multalin.toulouse.inra.fr/; free online) and/or BioEdit version 7.2 for mutation detection. Bar charts with prevalence of artemisinin resistance markers were generated using GraPhpad Prism 8.01 software. In order to predict the potential impact of non-synonymous SNPs on protein function, we used SIFT software (Sorting Intolerant From Tolerant, free online, https://sift.bii.a-star.edu.sg/index.html). SIFT software, takes into account the position at which the variation takes place and the type of amino acid change, then, chooses related proteins and obtains an alignment of these proteins with the query [34]. Finally, it calculates the probability that this particular amino acid change will is tolerated [34]. If the calculated value is less than a cutoff of 0.05, the substitution is predicted to be deleterious, and the opposite is considered not deleterious [34–36].

## Results

### Study participants

From the 450 participants included in the study, one third were from each of the three study provinces (Fig. 1). Most of the participants were males, representing 57, 53% (259/450). The age ranged from 6 months to 74 years, and the overall majority (%) were children between 1 to 12 years old, and the average was 15 years old.

### Artemisinin resistance *pfk13* polymorphism profile

From selected samples, 66, 2% (298/450) were confirmed for *P. falciparum* by real time PCR, and DNA was successfully sequenced for *pfk13*, where 98, 100 and 100 were from Niassa, Manica and Maputo, respectively (Table 1). No validated SNP for artemisinin derivate resistance was detected in our samples. However, in Niassa, 10.2% (9/98) of samples harbored nine different non-synonymous mutations (Fig. 1B, Table 1). In Manica and Maputo, five different non-synonymous mutations were detected in 6 (6/100) and 5 (5/100) samples, respectively (Fig. 1C, D). 56.3% (9/16) of the non-synonymous mutations reported in this study were by substitution at the 1st base of the codon, 25% (4/16) at the second base and 18.8% (3/16) at the third base. The non-synonymous mutation, W470R, was detected in all provinces and its prevalence decreases from north to south with 3%, 2% and 1% in Niassa, Manica and Maputo, respectively. Synonymous mutations were only detected in samples from Manica and Maputo (Fig. 1E).

**Table 1:** Single nucleotide polymorphisms identified in pfk13 gene in samples collected in Niassa, Manica and Maputo, Mozambique duiring 2021

Codon	Type of mutation	Reference» mutant	Study site	n/N
G449R	NS	ggt» **c**gt	Manica	1/98
V454E	NS	gta» g**a**a	Niassa	1/100
E455Q	NS	gaa» **c**aa	Maputo	1/100
S459S	S	tcg» tc**c**	Manica	3/98
D464Y	NS	gat» **c**at	Manica	1/98
D464H	NS	gat» **c**at	Niassa	1/100
C469C*	S	tgc» tg**t**	Manica	3/98
			Maputo	2/100
W470R	NS	tgg» **a**gg	Manica	2/98
			Niassa	3/100
			Maputo	1/100
V494L	NS	gtt» **c**tt	Niassa	1/100
E509D	NS	aga» a**a**a	Manica	1/98
C532W	NS	tgt» tg**g**	Niassa	1/100
I543S	NS	att» a**g**t	Maputo	1/100
G548G	S	ggg» gg**t**	Manica	1/98
G549G	S	tct» t**t**t	Manica	1/98
A578S	NS	gct» **t**ct	Niassa	1/100
A578P	NS	gct» **c**ct	Niassa	1/100
V581A	NS	gtt» g**c**t	Niassa	1/100
L598L	S	tta» **c**ta	Manica	1/98
Q654H	NS	caa» ca**c**	Manica	1/98
Q661H*	NS	caa» ca**c**	Maputo	1/100
L663I	NS	caa» ca**c**	Maputo	1/100

n: number of samples containing mutant allele; N: number of samples sequenced at locus; NS: non-synonymous SNP; S: synonymous SNP; * has previously been reported

### Putative functional impact of amino acid substitutions caused by the non-synonymous SNPs

With a cut-off score of 0.05, we predicted that V454E, V494L, A578P, V581A, E509D, E455Q and L663I mutations would be tolerated or have a non-deleterious effect on protein function, with equal score or higher than 0.05 as shown in the Fig. 1E. For the remaining SNPs, were predicted to affect protein function [34–36] with a score of 0.00 (Fig. 1E).

## Discussion

In Mozambique, the first-line for uncomplicated malaria treatment is AL and ASAQ the second-line and an alternative in case of contraindications [17]. Surveillance of validated and/or resistance-associated SNPs of the aforementioned artemisinin-based combinations is a powerful weapon to control the spread of parasite resistance through early detection of mutations [37–39]. Moreover, these mutations are being associated with the reduction in *pfkelch13* function, a protein required for parasite-mediated endocytosis of host haemoglobin in the newly invaded intra-erythrocytic ring stages [11, 39].

No validated mutations [6] for artemisinin resistance was observed from this study samples. Furthermore, 16 non-synonymous and 5 synonymous mutations were detected (Table 1). However, out of these 21 point mutations, four have been previously reported in Africa, namely C469C [40, 41], A578S [41–43], Q661H [41, 44], C532W [45] and one mentioned as candidate marker; at codon 449. Although this study observed a G449R instead of G449A as reported by the WHO [46]. The remaining 16-point mutations are reported here for the first time. In Mozambique, two other non-synonym mutations have been detected before, V494I in two samples from Maputo [29] and F656I in one sample (location not specified) [30]. In Africa, six non-synonymous SNPs validated as markers of artemisinin resistance, have been reported M476I, P553L, R561H, P574L, C580Y and A675V [22, 24, 26, 27, 45, 47]. In Rwanda, another SNPs were also detected P667S, Q661E [27], V55A, C532W and G533A [45]. Patients infected with Q661H or P667S had a Parasite Clearance Half-life > 5 h [27]. Q661H has not yet been validated as a marker yet, but the fact that it has been identified in more than one region requires closer monitoring. Although these results illustrate an emergence of resistance cases in Africa, the non-synonymous mutations reported in this study is not enough to raise an alarm in Africa as well as in Mozambique. Furthermore, the latest therapeutic efficacy study in Mozambique confirmed the susceptibility of *P. falciparum* to the drugs currently in use [19], however, continuous surveillance of ACT resistance markers is urgently needed.

The functionality of the protein was predicted. Thus, the obtained SIFT scores (Fig. 1) for V454E, V494L, A578P, V581A, E509D, E455Q and L663I indicate that these mutations are tolerated or have non-deleterious effect on protein function, because they are equal or above the cut off value of 0.05 [34–36]. For the remaining non-synonymous mutations, SIFT scores predicted them to be deleterious, with a score of 0.00, bellow cut off value of 0.05 [34–36]. SIFT issued a warning stating that there is low confidence in this prediction, since these substitutions may have been predicted to be deleterious just because the sequences used were not diverse enough [34].

These results showed that we do not have an emergency of artemisinin resistance cases, even though novel non-synonymous mutations were observed, highlighting the relevance of increasing the number of studies focused on the molecular surveillance of resistance markers to ACT.

## Conclusions

- No *pfkelch13*-mediated artemisinin resistance validated gene mutation was found from this study samples. Non-synonymous mutations were detected in all three provinces, with a prevalence of 11.22%, 6% and 5% in Niassa, Manica and Maputo, respectively.
- These results are limited to the selected provinces and districts. Hence the need to replicate these experiences and always with the aim of better informing PNCM decision-making and ultimately the genomics of malaria in Mozambique to become a programmatic activity. Further research is currently being done and will provide more information on the resistance profile to *Pfkelch13* [48].

## Data Availability

The data and materials analysed in this study are available from the corresponding author.
